# Educational and Employment Outcomes among Young Australians with a History of Depressive Symptoms: A Prospective Cohort Study

**DOI:** 10.3390/ijerph18073376

**Published:** 2021-03-24

**Authors:** Katrina Witt, Allison Milner, Tracy Evans-Whipp, John W. Toumbourou, George Patton, Anthony D. LaMontagne

**Affiliations:** 1 Turning Point, Eastern Health Clinical School, Monash University, Richmond 3121, Australia; katrina.witt@orygen.org.au; 2 Centre for Health Equity, Melbourne School of Population and Global Health, The University of Melbourne, Parkville 3052, Australia; allison.milner@unimelb.edu.au; 3 Centre for Adolescent Health, Murdoch Children’s Research Institute, Parkville 3052, Australia; tracy.evans@unimelb.edu.au (T.E.-W.); george.patton@rch.org.au (G.P.); 4 Department of Paediatrics, Royal Children’s Hospital and The University of Melbourne, Parkville 3052, Australia; 5 School of Psychology, Deakin University, Geelong 3220, Australia; john.toumbourou@deakin.edu.au; 6 Institute for Health Transformation and School of Health and Social Development, Deakin University, Geelong 3220, Australia

**Keywords:** depression, education, employment, psychosocial job quality

## Abstract

The aim of this study was to investigate whether depressive symptoms reported during adolescence are associated with subsequent educational and employment outcomes, including whether experiences of depressive symptoms in adolescence are associated with higher exposures to adverse psychosocial job stressors among those who were employed in emerging adulthood. We used data from the Victorian arm of the International Youth Development Study (IYDS). Multiple logistic regression analyses were used to model the association of depressive symptoms reported in 2002 (wave one) and/or 2003 (wave two) and self-reported completion of compulsory secondary schooling, employment status, and exposure to a number of psychosocial job stressors roughly a decade later (i.e., at wave three in 2014). In fully adjusted models, reporting high depressive symptoms at waves one or two (odds ratio (OR) 0.71, 95% confidence interval (CI) 0.55 to 0.92), as well as at both waves (OR 0.55, 95% CI 0.41 to 0.75) were associated with a reduced likelihood of completing secondary schooling by wave three. High depressive symptoms reported at multiple waves were also associated with a reduced likelihood of employment (OR 0.49, 95% CI 0.36 to 0.66). Amongst those employed at wave three (*n* = 2091; 72.5%), adolescent depressive symptoms were associated only with workplace incivility. Psychosocial job stressor exposures should be considered in the design and selection of jobs for young workers with a history of depressive symptoms in order to increase employment participation and sustainability for young people experiencing symptoms of depression.

## 1. Introduction

Adolescence is a critical developmental period during which many young people complete compulsory, or secondary, education and transition into further training, education, or employment [1]. The experience of mental health problems in adolescence can disrupt this process. Those who reported common mental health problems in adolescence were more likely to not be in employment, education, or training (NEET) as young adults [2]. This association has been observed even following adjustment for common confounders [3]. Even sub-threshold mental health problems experienced during adolescence have been associated with an increased risk of non-completion of compulsory schooling and unemployment [4].

However, far less is known about what happens at work among those young people with a history of mental health problems who do obtain paid work. While some work has found young people with a history of mental health problems may experience reduced wage earnings [5], to date only one study has investigated the longitudinal association of adolescent-onset mental illness on psychosocial job quality. A recent study of a large French working population sample found that self-reported adolescent-onset mental health problems were associated with reporting low predictability at work (i.e., predictability of time schedules and tasks), experiencing tension with the public, work-life imbalance, and shift-work particularly for males [6]. Moreover, these associations were stronger for younger workers (aged ≤ 30 years) as compared to older workers.

To the extent that people with a history of mental health problems may experience higher exposures to job stressors, this will be of concern with respect to the risk of relapse or further mental health problems and the sustainability of employment. Exposure to adverse job stressors has been found to increase the risk of depression, anxiety, and other mental health problems in general working population samples [7,8,9,10]. With growing policy and practice interest in promoting employment for people with past or current mental health problems [11,12,13], a better understanding of working conditions in this context could inform the selection and design of jobs to improve employment participation and sustainability for this group.

To address this gap in the literature, we sought to investigate the association between depressive symptoms in adolescence and later educational and employment outcomes in a representative cohort of young Australians. In particular, we sought to extend this investigation to follow young people from adolescence into employment, and to assess whether a history of depressive symptoms in adolescence is associated with exposure to mental health-averse psychosocial job stressors. Specifically, we hypothesized that a history of depressive symptoms reported during adolescence will be associated with: [1] lower educational attainment; [2] reduced odds of employment; and [3] greater exposure to psychosocial job stressors among those who obtain employment in emerging adulthood approximately a decade later. Given recent findings that experiencing symptoms of depressive symptoms throughout adolescence has a stronger association with educational and employment outcomes [3], we further hypothesized that depressive symptoms reported at one adolescent wave will only be associated with a weaker association on educational and employment outcomes as compared to depressive symptoms experienced at both adolescent survey waves.

## 2. Materials and Methods

Data were obtained from the International Youth Development Study (IYDS), an ongoing multiwave longitudinal survey of young people living in the state of Victoria, Australia and Washington State, USA. For this study, only data from the Victorian arm of the IYDS were used to investigate prospective associations between depressive symptoms in adolescence and educational and employment outcomes in young adulthood.

A population-based cohort of Victorian school students were recruited into the original IYDS survey in 2002. Information on the way in which schools and adolescents were selected for participation is described in greater detail elsewhere [14]. In brief, a two-stage cluster approach was used to approximate a state representative sample of school students. At wave one of the IYDS, when respondents were between 11.3 and 14.2 years of age, sociodemographic factors, depressive symptoms, and alcohol and drug use were assessed. Respondents were re-interviewed capturing the same measures one year later in 2003, when participants were between 12.2 to 15.6 years (wave two).

Roughly a decade later, in 2014, a total of 1689 respondents (86.3% of the original cohort) were again contacted to provide data on sociodemographic factors, depressive symptoms and alcohol use (wave three). As respondents were also between 23.3 to 26.6 years of age at this survey wave, further items on adult transitions, including educational attainment, employment status, and information on psychosocial job quality for those who indicated they were currently employed were also included.

### 2.1. Exposure Variables

#### Depressive Symptoms during Adolescence

Scores on the self-reported Short Mood and Feelings Questionnaire (SMFQ) [15] were used to measure depressive symptoms at both waves one and two of the IYDS survey. The SMFQ is a 13-item subscale, formed from the longer 33-item Mood and Feelings Questionnaire (MFQ), in which respondents are asked to indicate the frequency of experience of a number of depressive symptoms consistent with Diagnostic and Statistical Manual of Mental Disorders (DSM) criteria for major depression over the past two weeks. Respondents rate each statement on a three-point Likert scale ranging from zero (“not true”) to two (“often true”), with higher scores indicative of greater levels of depressive symptomatology [16]. Cronbach’s alpha for the SMFQ was 0.86 for wave one and 0.91 for wave two [17], indicating satisfactory internal reliability. The SMFQ has good criterion validity when assessed against the Diagnostic Interview Schedule for Children (DISC) depression scale [15,18], the International Classification of Disease-version 10 (ICD-10), and the DSM-fourth revision (DSM-IV) [19], and has previously been used in the IYDS to indicate depression “caseness” [16,20]. For the purposes of this study, a score of 11 or greater on the SMFQ was used to define high depression symptoms, while a score of 8 or greater but less than 11 on the SMFQ was used to define moderate depression symptoms. We further investigated the association of depression symptoms experienced at either wave one, two, or at both adolescent waves of the IYDS.

### 2.2. Outcome Variables

We extracted data on employment status, completion of secondary education, completion of tertiary education, and current employment from the third wave of the IYDS survey, when respondents were between 21.9 and 28.9 years of age (median: 25.0 years; interquartile range (IQR): 23.3 to 26.6 years).

#### 2.2.1. Completion of Secondary Education

At wave three, respondents were asked: “What is the highest year level at secondary school you completed?”. Response options could range from “Year 8 or below”; “Year 9 or equivalent”; “Year 10 or equivalent”; “Year 11 or equivalent”; and “Year 12 or equivalent”. Those respondents indicating they had completed year 12 or equivalent (indicating completion of secondary education) formed the comparison group, whilst those indicating they had completed less than 12 years of schooling formed the reference group [21].

#### 2.2.2. Completion of Tertiary Education

Respondents were also asked: “What is the highest level of education you have completed, since secondary schooling?” with response options ranging from “I have not completed any study since secondary school”; to “apprenticeship/traineeship”; “certificate course (certificate I, II, III, or IV)”; “graduate diploma/certificate”; “advance diploma/certificate”; “bachelor-level degree”; to “postgraduate degree”. Respondents indicating they had either completed or were completing any apprenticeship/traineeship, any certificate course, any diploma, or any bachelor or postgraduate degree formed the reference group, whilst those indicating they had not completed any further study since secondary school formed the comparison group [21].

#### 2.2.3. Obtaining Paid Employment

Responses to the question “Are you currently employed?” were coded dichotomously, with all respondents indicating they were not employed (including those who indicated they were unemployed, were in education or training, not in the labor force, as well as those not in education, employment, or training) forming the comparison group and those reporting they were currently employed the reference group [21]. Data on current employment status (at wave 3) was missing for 370 respondents (12.8 %).

### 2.3. Perceived Psychosocial Job Stressors

For those respondents who indicated they were employed at the third survey wave, a series of questions asked about exposure to psychosocial job stressors. Responses to each of these questions were made on a four-point Likert scale ranging from one (“strongly agree”) to four (“strongly disagree”). These measures are detailed in turn below.

#### 2.3.1. Psychosocial Job Demands

This domain measures the quantitative psychological demands an individual experiences [22] and consisted of the sum of the following three items: “I am not asked to do an excessive amount of work”; “I have enough time to get the job done”; and “I am free from the conflicting demands of others”, with higher values indicating higher perceived psychological job demands (Cronbach’s alpha = 0.76). We defined high psychological job demands (i.e., adverse exposure) by values above the median.

#### 2.3.2. Skill Discretion

Given previous research distinguishing skill discretion from decision authority [23], we examined these constructs separately. The skill discretion domain measures the degree to which a job involves a variety of tasks, low levels of repetitiveness, and opportunities for creativity and skill development [22] and was formed from the sum of the following five items: “My job requires that I learn new things”; “My job requires me to be creative”; “My job requires a high level of skill”; “I get to do a variety of things at my job”; and “I have an opportunity to develop my own special abilities at work” with higher values indicating lower perceived skill discretion (Cronbach’s alpha = 0.87). We defined low skill discretion (i.e., adverse exposure) by values above the median.

#### 2.3.3. Decision Authority

The decision authority domain measures the degree to which an individual has control over the way in which they complete tasks [22] and was formed from the sum of the following three items, assessed at wave three: “My job allows me to make a lot of decisions on my own”; “I am given a lot of freedom to decide how I do my job”; and “I have a lot to say about what happens at my job”, with higher values indicative of lower decision authority (Cronbach’s alpha = 0.86). We defined low decision authority (i.e., adverse exposure) by values above the median.

#### 2.3.4. Job Control

This domain measures the degree to which an individual has control over their tasks [22] and was formed by equally weighting the five items from the skill discretion domain and the three items from the decision authority domain to form a measure of job control, with higher values indicating lower perceived job control (Cronbach’s alpha = 0.90). We defined low job control (i.e., adverse exposure) by values above the median.

#### 2.3.5. Job Strain

Job strain (i.e., adverse exposure) was defined as the co-occurrence of high psychological job demands and low job control.

#### 2.3.6. Workplace Incivility

Following previous research [24], a measure of incivility in the workplace was formed by combining responses from the following three items: “In the past six months, how often have you been ignored or excluded at work?”; “How often have you been humiliated or ridiculed at work?”; and “How often has someone withheld information that affected your performance?”. Responses to these three items were made on a two-point Likert scale ranging from zero (“never”) to one (“at least once”) Respondents indicating exposure to any one of these three stressors on at least one occasion over the past month were recorded as having experienced workplace incivility (Cronbach’s alpha = 0.80).

### 2.4. Potential Confounders

We adjusted for measured variables that could plausibly constitute prior common causes of both the exposure variables in adolescence (i.e., depressive symptoms) and each of the outcomes in young adulthood and as informed by previous work. These included gender, parental sociodemographic status, and age (all reported at wave one).

#### Parental Sociodemographic Status

Parental socio-demographic status was derived as a composite measure including the respondent’s parental educational level and income as reported by respondents’ parents at wave one. This composite measure could take any value between one and three, with higher values indicative of higher parental socio-demographic status. The scores were then converted into quartiles [25].

### 2.5. Statistical Analyses

Multiple logistic regression analyses were used to model the association of depression symptoms reported at wave one, two, or both waves on: (1) completion of compulsory secondary education; (2) employment status at wave three; and (3) perceived exposure to psychological job demands, skill discretion, decision authority, job control, job strain, and workplace incivility at wave three.

Two models were presented for each outcome: (1) unadjusted associations; and (2) following adjustment for covariates. All analyses were undertaken in Stata for Windows, version 14 [26].

## 3. Results

At wave one, a total of 2884 Victorian school children between the ages of 10.0 to 16.5 years (median: 12.9 years; IQR: 11.3 to 14.6 years) responded to the IYDS survey. All 2884 respondents were followed up one year later at wave two when they were between the ages of 11.0 to 17.4 years (median: 14.0 years; IQR: 12.2 to 15.6 years). At wave three, 2518 (87.3% of the original sample) of the original respondents were followed-up when they were between 21.9 years and 28.9 years (median: 25.0 years; IQR: 23.3 to 26.6 years).

Almost one-half of the sample was female. At wave one, one in five were in the lowest quartile for family socio-economic status (SES) in adolescence whilst almost one quarter were in the highest quartile (*n* = 622; 21.6% vs. *n* = 682; 23.6%). For 203 (7.0%) respondents, data on the SMFQ were missing at either waves one or two. Further characteristics of respondents at waves one, two, and three are provided in Table 1.

### 3.1. Completion of Compulsory Secondary Schooling

A total of 1969 (68.3%) respondents indicated they had completed secondary schooling by wave three of the IYDS survey (852 males and 1117 females); however, responses to this item were missing for 372 (12.9%) respondents. In fully adjusted models, high depressive symptoms reported at either waves one or two was associated with a 29% reduced odds of completing secondary schooling, whilst high depressive symptoms reported at both survey waves were associated with approximately 45% reduced odds of completing secondary schooling by wave three (Table 2). The magnitude of association for moderate depression symptoms reported at either waves one or two were similar; however, there was no significant association for moderate depression symptoms reported at both adolescent survey waves and the likelihood of completing compulsory secondary schooling by young adulthood.

### 3.2. Obtaining Paid Employment

By wave three of the IYDS survey, 2091 (72.5%) respondents indicated they were currently employed. Of these, 1094 (52.3%) were female and 997 (47.7%) were male. In fully adjusted models, high depressive symptoms reported at both waves were associated with a 51% reduced odds of reporting current employment at wave three (Table 2). There were no significant associations between reporting moderate depressive symptoms and the likelihood of being currently employed in young adulthood.

### 3.3. Associations with Percieved Psychosocial Job Stressors

All subsequent analyses are restricted to the 2091 respondents that reported they were currently employed by wave three.

#### 3.3.1. Psychosocial Job Demands

A total of 656 (31.4%) respondents indicated they were exposed to high psychological demands in their current role; 374 (57.0%) were females and 282 (43.0%) were males. In unadjusted models, reporting high depressive symptoms at both waves one and two were both associated with greater perceived psychological job demands; however, following adjustment for gender, family SES in adolescence, and age these associations were attenuated and confidence intervals no longer excluded the null value (Table 3). There were also no significant associations for reporting moderate depression symptoms and later experiences of high psychological job demands in young adulthood.

#### 3.3.2. Low Skill Discretion

A total of 966 (46.2%) respondents indicated experiencing low skill discretion at work. There were no significant associations for either high or moderate symptoms of depression, reported either at wave one, two, or both survey waves, and low skill discretion at wave three (Table 3).

#### 3.3.3. Low Decision Authority

There were no statistically significant associations between either high or moderate depression symptoms reported at waves one, two or both waves and low decision authority in either unadjusted or adjusted models for the 841 (40.2%) of respondents indicating they experienced low decision authority at work (Table 3).

#### 3.3.4. Low Job Control

A total of 815 (39.0%) of respondents indicated they experienced low job control at work at wave three. Neither high nor moderate symptoms of depression reported at waves one, two, or at both waves were associated with low job control in either unadjusted or adjusted models (Table 3).

#### 3.3.5. High Job Strain

A total of 295 (14.1%) of respondents indicated they were exposed to high job strain at work. In univariate models, high symptoms of depression reported at both waves one and two was associated with an increased likelihood of experiencing high job strain at work. However, following adjustment for gender, family SES during adolescence, and age the association was attenuated and the confidence interval included the null (Table 3). There were additionally no significant associations between reporting moderate symptoms of depression at either waves one, two, or at both survey waves and the likelihood of experiencing high job strain in young adulthood.

#### 3.3.6. Workplace Incivility

Just under one-half (*n* = 988; 47.2%) of respondents indicated they had been exposed to incivility in the workplace over the past six months. In adjusted models, high symptoms of depression reported at waves one or two were associated with a 26% increased odds of reporting workplace incivility at work, with symptoms of depression reported at both survey waves associated with approximately 70% increased odds of reporting exposure to workplace incivility (Table 3). The experience of moderate levels of depression in adolescence was also associated with a 25% increased likelihood of reporting experiences of workplace incivility in the past six months; however, reporting moderate levels of depression at both survey waves was not associated with a significantly increased likelihood of reporting exposure to workplace incivility in this study.

### 3.4. Sensitivity Analyses

We also undertook sensitivity analyses using a cut-off of 12 on the SFMQ. There were no material differences to our results for any of the educational, employment, or perceived psychosocial job stressors outcomes as reported in this study using this revised cut-point.

## 4. Discussion

This study is one of few internationally to investigate not only educational and employment outcomes following adolescent-onset depression, but also the association between adolescent history of depression and subsequent working conditions during young adulthood. Whilst the majority of the cohort went on to obtain paid employment, our findings did demonstrate that an adolescent history of depression symptoms predicted lower educational attainment and lower likelihood of employment in emergent adulthood in a population-based cohort of 2518 young Australians.

We also found that, amongst those 2091 respondents who indicated they were currently employed in young adulthood, a history of depression during adolescence was not associated with an increased likelihood of reporting higher job demands, higher job strain, or exposure to workplace incivility. After adjustment for common prior confounders, however, there was a modest prospective association between adolescent depression and exposure to workplace incivility. Thus our hypothesized prediction of higher exposure to job stressors among those with a history of depressive symptoms was only partially confirmed. A previous French survey of the working-age population also found associations between having a history of self-reported diagnoses of mental illnesses and poorer educational, employment, and income outcomes [27]. However, this study also found associations between self-reported history of depression and greater exposure to a greater number of psychosocial job stressors [6], in contrast to the present study. This may be explained by between-country differences, or differences in the measures and methods used.

A history of adolescent-onset depression could lead to greater exposure to psychosocial job stressors in a number of ways. For example, work has previously shown that reduced educational attainment has been associated with employment in lower occupational skill level jobs which, is turn, has been associated with higher exposure to job stressors [28]. Reduced and/or disrupted work experience due to mental health problems may also predispose some young people to employment in jobs characterized by lower occupational skill levels. Finally, discrimination on the basis of mental health history could also result in young people with this history having to take jobs characterized by poorer psychosocial quality. However, alternative explanations remain to be ruled out in future research. For example, other processes linked to a higher likelihood of experiencing depressive symptoms in adolescence could also eventually contribute to early school termination, such as experiencing bullying in childhood or emerging academic difficulties. So too, different experiences in the workplace might reflect the persistence of earlier risks (e.g., being bullied at work), or might be a consequence of persisting depressive symptoms interfering with social interaction, or a heightened sensitivity to criticism that occurs in the workplace as a result of continuing depressive mood state. It is also important to note that the earlier study [6] was conducted among the general working age population, rather than being restricted to young people only.

Future research should also investigate the prospective association of adolescent-onset self-harm and psychosocial job quality in young adulthood given recent research which found that young adults reporting a history of self-harm during adolescence are less likely to complete secondary schooling [29,30,31], are more likely to be report experiencing longer periods of unemployment [32,33,34], and/or NEET [29], and are more likely to have lower grade point averages in their first year of tertiary education as compared to their peers [35]. Whilst we had intended to investigate these associations, data on self-reported self-harm were missing for almost half of IYDS respondents (*n* = 1236; 42.9%), precluding meaningful analysis in this study.

### 4.1. Limitations

Firstly, the determination of depressive symptoms in this study was based on a cut-off score of 11 or higher on the SMFQ, as has previously been used in the IYDS to indicate depression ‘caseness’ [16,20]. Sensitivity analyses using an alternative cut-off point of 12, as has been used in some other work, did not materially affect our findings. Whilst the SMFQ has been previously used to indicate potentially diagnosable levels of depression in adolescents [20,36], the SMFQ items focus predominately on symptoms of depressed mood and anhedonia; the two core symptoms of ICD and DSM depression. Whilst these two symptom domains have been found to have high predictive value for depression diagnoses in adulthood, other relevant symptom constructs, such as feelings of worthlessness [37], and feelings of being unloved [36], are not assessed by the SMFQ. Additionally, whilst the prevalence of depressive symptoms found in this study is similar to that found in other international work using the SMFQ to ascertain depression caseness [38], the prevalence of those reporting depression caseness in this study is significantly higher than estimates obtained from diagnostic information [39]. The assessment of depressive symptoms in this study also covered the previous two weeks. It is likely that some episodes of moderate or high depressive symptoms may have been missed, therefore resulting in more conservative estimates.

Additionally, we only had a simplistic measure of employment, limiting our ability to explore associations between adolescent experiences of depressive symptoms and employment type (e.g., temporary versus permanent) and further employment characteristics (e.g., part-time versus full-time, underemployment). Further, to parallel our outcomes of completing secondary and tertiary education, our analysis focused on the positive outcome of obtaining paid employment. We combined those not in the labor force (NILF) due to disability, caring responsibilities or other reasons and those not in education, employment, or training (NEET) in the alternate category of ‘not employed.’ Hence some of those classified as not employed in the present study might nonetheless otherwise be constructively engaged in caring roles and/or in undertaking full-time study or training.

We used median splits in the present study for each of the psychosocial job stressors investigated, as is standard in this field. Whilst a multinomial design may have given us greater discriminatory power to investigate associations between adolescent-onset depressive symptoms and each of the psychosocial job stressors investigated, these analyses would have been more difficult to interpret and less conservative. Further, our measures of psychosocial job stressors were limited to only a few; others may be important as well, such as job insecurity, social support at work, and bullying (though our measure of incivility likely overlaps to some extent with bullying).

Other important confounding factors for the association between depressive symptoms and both educational and employment outcomes in adulthood may exist (e.g., experience of adverse events in childhood, cognitive functioning, etc.). However, we were unable to include such factors in our models as data were unavailable in the IYDS cohort. The above limitations are offset by particular strengths of the study: its higher generalizability due to its population-based sample, the longitudinal design and coverage of the transition between adolescence/education and employment in young adulthood (more than a decade), and the high follow-up achieved (86% at Wave 3). Finally, we had moderate loss to follow-up (14%) and based our results on complete case analyses.

### 4.2. Implications for Policy and Practice

To date the focus on improving employment participation in those with depressive symptoms and other mental health problems has been to develop and evaluate supported employment interventions to improve employment participation rates in those recovering from mental illness. These programs have, to date, given little consideration to job quality. Results from this study, together with other recent international work [6], would suggest that employment quality should be an important consideration both in the selection and design of jobs for workers with current or past history of depression or other mental health problems. Consideration of the psychosocial characteristics of jobs should be part of job selection in the open employment market, and should also be part of job design for supported employment policies and procedures for young adults with a history of mental health problems. Towards this end, population-based ‘job-exposure matrices’ could be used to characterize the typical psychosocial job characteristics associated with different job titles, providing a source of information for policy-makers and practitioners engaged in the improvement of employment participation and sustainability for the growing population group with a history of mental health problems [40].

We believe that further research into the psychosocial quality of work is important, as almost three-quarters of cohort participants with a history of depression in adolescence were in employment by wave three. Additionally, given the increasing interest in functional recovery programs, and particularly in individual placement and support (IPS) programs, to assist young people with mental health problems to transition into employment, we believe the findings of our study suggest that the psychosocial quality of these placements may be important in determining the success of these programs, and should be considered as a factor in open employment for people with a history of depression in adolescence.

## 5. Conclusions

This study confirmed previously documented associations between adolescent history of depression and lower educational attainment and employment in young adulthood, but importantly, extended this work to show that adolescent experiences of depression were also associated with an increased risk of reporting workplace incivility among those who were employed a decade later. Given that exposure to poor psychosocial working conditions is associated with deterioration in mental health, policies aimed at increasing workforce participation of young people, and particularly those with a history of mental illness, should attend to the psychosocial quality of employment to ensure that employment in the open labor market or through vocational rehabilitation helps, rather than hinders, in the recovery from mental illness. The Australian experience, as represented in this study, suggests that youth with a history of mental health problems who do obtain employment in young adulthood end up, for the most part, in jobs with acceptable psychosocial working conditions, whereas the opposite was the case in the only other such study to date in France [6]. Whether this is attributable to Australian policy of practice could be investigated in future studies.

## Figures and Tables

**Table 1:** Characteristics of the respondents at the adolescent (one and two) and young adulthood (three) waves of the International Youth Development Study (IYDS).

Characteristic		WAVE 1		WAVE 2		WAVE 3	
		Md	IQR	Md	IQR	Md	IQR
Age in years		12.9	(11.3 to 14.6)	14.0	(12.2 to 15.6)	25.0	(23.3 to 26.6)
		**M**	**SD**	**M**	**SD**	**M**	**SD**
Depression symptoms (SMFQ)		7.0	5.3	6.9	6.1	-	-
Percieved psychosocial job stressors							
	*Job demands*		-		-	6.8	1.8
	*Skill discretion*		-		-	9.5	3.4
	*Decision authority*		-		-	6.2	2.3
	*Job control*		-		-	78.4	25.6
		***N* (%)**		***N* (%)**		***N* (%)**	
Gender							
	*Male*	1394 (48.3)		1395 (48.4)		1157 (40.1)	
	*Female*	1490 (51.7)		1489 (51.6)		1360 (47.2)	
	*Missing*	0 (0.0)		0 (0.0)		367 (12.7)	
Family socio-economic status in adolescence							
	*Lowest quartile*	622 (21.6)		-		-	
	*Highest quartile*	682 (23.6)		-		-	
	*Missing*	230 (8.0)		-		-	
High depression symptoms (SMFQ ≥ 11)							
	*Yes*	637 (22.1)		660 (22.9)		-	
	*No*	2134 (74.0)		2122 (73.6)		-	
	*Missing*	113 (3.9)		102 (3.5)		-	
Moderate depression symptoms (SMFQ ≥ 8 but <11)							
	*Yes*	397 (13.8)		356 (12.3)		-	
	*No*	2374 (82.3)		2426 (84.1)		-	
	*Missing*	113 (3.9)		102 (3.5)		-	
Current employment status							
	*Employed*	-		-		2091 (72.5)	
	*Not employed*	-		-		423 (14.7)	
	*Missing*	-		-		370 (12.8)	

**Notes:** IQR—interquartile range; M—mean; Md—median; SD—standard deviation; SMFQ—Short Mood and Feelings Questionnaire.

**Table 2:** Prospective associations between symptoms of depression and educational and employment outcomes at wave three.

		Completion of Secondary Schooling		Current Employment	
		Unadjusted	Adjusted ^1^	Unadjusted	Adjusted ^1^
		OR (95% CI)	OR (95% CI)	OR (95% CI)	OR (95% CI)
No depression symptoms ^2^		1.00	1.00	1.00	1.00
High depression symptoms ^3^					
	*Waves one or two (N = 958)*	**0.74 (0.59–0.94)**	**0.71 (0.55–0.92)**	0.85 (0.65–1.11)	0.90 (0.68–1.20)
	*Both waves one and two (N = 339)*	**0.56 (0.43–0.73)**	**0.55 (0.41–0.75)**	**0.49 (0.37–0.65)**	**0.49 (0.36–0.66)**
Moderate depression symptoms ^4^					
	*Waves one or two (N = 684)*	**0.74 (0.59–0.92)**	**0.74 (0.57–0.94)**	0.94 (0.73–1.21)	0.95 (0.72–1.24)
	*Both waves one and two (N = 69)*	1.00 (0.55–1.82)	1.35 (0.68–2.66)	1.33 (0.65–2.71)	1.38 (0.65–2.95)

**Notes:** CI—confidence interval; OR—odds ratio. Values in boldface significant at the *p* < 0.05 level. ^1^ Adjusted for gender (wave three), parental sociodemographic status, and age (both wave one); ^2^ Reference category; ^3^ Total score on the Short Mood and Feelings Questionnaire of 11 or greater; ^4^ Total score on the Short Mood and Feelings Questionnaire of 8 or greater, but less than 11.

**Table 3:** Prospective associations between symptoms of depression, and subsequent exposure to perceived psychosocial job domains in those currently employed at wave three (*n =* 2091).

		High Psychological Job Demands		Low Skill Discretion		Low Decision Authority		Low Job Control		High Job Strain		Workplace Incivility	
		Unadjusted	Adjusted ^1^	Unadjusted	Adjusted ^1^	Unadjusted	Adjusted	Unadjusted	Adjusted	Unadjusted	Adjusted	Unadjusted	Adjusted
		OR (95% CI)	OR (95% CI)	OR (95% CI)	OR (95% CI)	OR (95% CI)	OR (95% CI)	OR (95% CI)	OR (95% CI)	OR (95% CI)	OR (95% CI)	OR (95% CI)	OR (95% CI)
No depressive symptoms(either wave) ^2^		1.00	1.00	1.00	1.00	1.00	1.00	1.00	1.00	1.00	1.00	1.00	1.00
High depression symptoms ^3^													
	Waves 1 or 2	1.06 (0.84–1.34)	1.01 (0.79–1.28)	0.99 (0.80–1.23)	1.01 (0.81–1.26)	0.84 (0.67–1.05)	0.83 (0.66–1.04)	0.94 (0.75–1.17)	0.97 (0.77–1.22)	0.94 (0.68–1.29)	0.94 (0.68–1.31)	**1.25** **(1.01–1.55)**	**1.26** **(1.01–1.58)**
	Both waves 1 and 2	**1.50** **(1.13–1.20)**	1.28 (0.94–1.73)	0.93 (0.71–1.23)	0.94 (0.70–1.26)	1.03 (0.78–1.37)	0.94 (0.70–1.27)	1.02 (0.77–1.35)	0.99 (0.74–1.34)	**1.59** **(1.11–2.27)**	1.38 (0.94–2.01)	**1.81** **(1.36–2.39)**	**1.70** **(1.26–2.28)**
Moderate depressionsymptoms ^4^													
	Waves 1 or 2	0.98 (0.78–1.23)	1.02 (0.81–1.30)	1.07 (0.87–1.32)	1.07 (0.86–1.33)	1.07 (0.87–1.33)	1.09 (0.89–1.39)	1.14 (0.92–1.41)	1.17 (0.94–1.50)	0.80 (0.58–1.10)	0.84 (0.61–1.17)	1.21 (0.98–1.50)	**1.25** **(1.00–1.56)**
	Both waves 1 and 2	1.09 (0.63–1.89)	1.07 (0.60–1.92)	0.90 (0.54–1.52)	0.81 (0.47–1.40)	1.43 (0.85–2.40)	1.50 (0.87–2.58)	1.16 (0.69–1.95)	1.13 (0.65–1.96)	0.90 (0.42–1.91)	0.95 (0.44–2.05)	1.02 (0.61–1.70)	1.05 (0.61–1.81)

**Notes:** CI—confidence interval; OR—odds ratio. Values in boldface are significant at the *p* < 0.05 level. ^1^ Adjusted for gender (wave three), parental sociodemographic status (wave one), and age (wave one). ^2^ Reference category. ^3^ Total score on the Short Mood and Feelings Questionnaire of 11 or greater. ^4^ Total score on the Short Mood and Feelings Questionnaire of 8 or greater, but less than 11.

## Data Availability

Data is available on reasonable request from the Centre for Adolescent Health, Murdoch Children’s Research Institute, Parkville, 3052, VIC, Australia.

## References

[1] Henderson M., Harvey S., Overland S. (2011). Work and common psychiatric disorders. J. R. Soc. Med..

[2] López-López J., Kwong A., Washbrook E., Pearson R., Tilling K., Fazel M., Kidger J., Hammerton G. (2019). Trajectories of depressive symptoms and adult educational and employment outcomes. BJPsych Open.

[3] Rodwell L., Romaniuk H., Nilsen W., Carlin J., Lee K., Patton G. (2018). Adolescent mental health and behavioural predictors of being NEET: A prospective study of employment iof young adults not in employment, education, or training. Psychol. Med..

[4] Copeland W., Wolke D., Shanahan L., Costello E. (2015). Adult functional outcomes of common childhood psychiatric problems: A prospective, longitudinal study. JAMA Psychiatry.

[5] Fletcher J. (2013). Adolescent depression and adult labor market outcomes. South. Econ. J..

[6] Witt K., Milner A., Chastang J., LaMontange A., Nidehammer I. (2018). Impact of lifetime compared to adolescent-onset mental illness on psychosocial employment quality in adulthood: Analysis of a nationally representative French cohort. Int. Arch. Occup. Environ. Health.

[7] Harvey S.B., Modini M., Joyce S., Milligan-Saville J.S., Tan L., Mykletun A., Bryant R.A., Christensen H., Mitchell P.B. (2017). Can work make you mentally ill? A systematic meta-review of work-related risk factors for common mental health problems. Occup. Emviron. Med..

[8] Madsen I.E., Nyberg S.T., Hanson L.M., Ferrie J.E., Ahola K., Alfredsson L., Batty G.D., Bjorner J.B., Borritz M., Burr H. (2017). Job strain as a risk factor for clinical depression: Systematic review and meta-analysis with additional individual participant data. Psychol. Med..

[9] Rugulies R., Aust B., Madsen I. (2017). Effort-reward imbalance at work and risk of depressive disorders. A systematic review and meta-analysis of prospective cohort studies. Scand. J. Work Environ. Health.

[10] Theorell T., Hammarström A., Aronsson G., Bendz L.T., Grape T., Hogstedt C., Marteinsdottir I., Skoog I., Hall C. (2015). A systematic review including meta-analysis of work enviornment and depressive symptoms. BMC Public Health.

[11] OECD (2015). Mental Health and Work: Australia.

[12] OECD (2012). Sick on the Job? Myths and Realities about Mental Health and Work.

[13] OECD (2008). Are All Jobs Good for Your Health? The Impact of Work Status and Working Conditions on Mental Health. OECD Employment Outlook, 2008.

[14] McMorris B., Hemphill S., Toumbourou J., Catalano R., Patton G. (2007). Prevalence of substance use and delinquent behavior in adolescents from Victoria, Australia and Washington State, United States. Health Educ. Behav..

[15] Angold A., Costello E., Messer S., Pickles A. (1995). Development of a short questionnaire for use in epidemiological studies of depression in children and adolescents: Factor composition and structure across development. Int. J. Methods Psychiatr. Res..

[16] Messer S., Angold A., Costello E., Loeber R., van Kammen W., Stouthamer-Loeber M. (1995). Development of a short questionnaire for use in epidemiological studies of depression in children and adolescents: Factor composition and structure across development. Int. J. Methods Psychiatr. Res..

[17] Lewis A., Rowland B., Tran A., Solomon R., Patton G., Catalano R., Toumbourou J. (2017). Adolescent depressive symptoms in India, Australia, and the USA: Exploratory structural equation modelling of cross-national invariance and predictions by gender and age. J. Affect. Disord..

[18] Rhew I., Simpson K., Tracy M., Lymp J., McCauley E., Tsuang D., van der Stoep A. (2010). Criterion validity of the Short Meed and Feelings Questionnaire and one- and two-item depression screens in young adolescents. Child Adolesc. Psychiatr. Ment. Health.

[19] Turner N., Joinson C., Peters T., Wiles N., Lewis G. (2014). Validity of the Short Mood and Feelings Questionnaire in late adolescence. Psychol. Assess..

[20] Patton G., Hemphill S., Beyers J., Bond L., Toumbourou J., McMorris B., Catalano R. (2007). Pubertal stage and deliberate self-harm in adolescents. J. Am. Acad. Child Adolesc. Psychiatry.

[21] Scholes-Balog K., Hemphill S., Evans-Whipp T., Toumbourou J., Patton G. (2016). Developmental trajectories of adolescent cannabis use and their relationship to young adult social behavioural adjustment: A longitudinal study of Australian youth. Addict. Behav..

[22] Karasek R. (1979). Job demands, job decision latitude, and mental strain: Implications for job redesign. Adm. Sci. Q..

[23] Karasek R., Brisson C., Kawakami N., Houtman I., Bongers P., Amick B. (1998). The Job Content Questionnaire (JCQ): An instrument for internationally comparative assessments of psychosocial job characteristics. J. Occup. Health Psychol..

[24] Cortina L., Lilia M., Magley V., Williams J., Langhout R. (2001). Incivility in the workplace: Incidence and impact. J. Occup. Health Psychol..

[25] Langford E. (2017). Quartiles in elementary statistics. J. Stat. Educ..

[26] StataCorp (2015). Stata Statistical Software: Release 14.

[27] Witt K., Milner A., Chastang J., LaMontange A., Niedhammer I. (2019). Employment and occupational outcomes following adolescent-onset mental illness: Analysis of a nationally representative French cohort. J. Public Health.

[28] LaMontagne A., Krnjacki L., Kavanagh A., Bentley R. (2013). Psychosocial working conditions in a representative sample of working Australians 2001-2008: An analysis of changes in inequalities over time. Occup. Environ. Med..

[29] Mars B., Heron J., Crane C., Hawton K., Lewis G., Macleod J., Tilling K., Gunnell D. (2014). Clinical and social outcomes of adolescent self harm: Population based birth cohort study. BMJ.

[30] Harrington R., Pickles A., Aglan A., Harrington V., Burroughs H., Kerfoot M. (2006). Early adult outcomes of adolescents who deliberately poisoned themselves. J. Am. Acad. Child Adolesc. Psychiatry.

[31] Brière F., Rohde P., Seeley J., Klien D., Lewinsohn P. (2014). Adolescent suicide attempts and adult adjustment. Depress. Anxiety.

[32] Borschmann R., Becker D., Coffey C., Spry E., Moreno-Betancur M., Moran P., Patton G. (2017). 20-year outcomes in adolescents who self-harm: A population-based cohort study. Lancet Child Adolesc. Health.

[33] Goldman-Mellor S., Caspi A., Harrington H., Hogan S., Nada-Raja S., Poulton R., Moffitt T. (2014). Suicide attempt in young people: A signal for long-term health care and social needs. JAMA Psychiatry.

[34] Niederkrotenthaler T., Tinghög P., Alexanderson K., Dahlin M., Wang M., Beckman K., Gould M., Mittendorfer-Rutz E. (2014). Future risk of labour market marginalization in young suicide attempters—A population-based prospective cohort study. Int. J. Epidemiol..

[35] Mortier P., Demyttenaere K., Auerbach R., Green J., Kessler R., Kiekens G., Nock M., Bruffaerts R. (2015). The impact of lifetime suicidality on academic performance in college freshmen. J. Affect. Disord..

[36] McKenzie D., Toumbourou J., Forbes A., Mackinnon A., McMorris B., Catalano R., Patton G. (2011). Predicting future depression in adolescents using the Short Mood and Feelings Questionnaire: A two-nation study. J. Affect. Disord..

[37] Wilcox H., Anthony J. (2004). Child and adolescent clinical features as forerunners of adult-onset major depressive disorder: Retrospective evidence from an epidemiological sample. J. Affect. Disord..

[38] Larsson B., Ingul J., Jozefiak T., Leikanger E., Sund A. (2016). Prevalence, stability, 1-year incidence and predictors of depressive symptoms among Norwegian adolescents in the general population as measured by the Short Mood and Feelings Questionnaire. Nord J. Psychiatry.

[39] Sawyer M., Reece C., Sawyer A., Johnson S., Lawrence D. (2018). Has the prevalence of child and adolescent mental disorders in Australia changed between 1998 and 2013 to 2014?. J. Am. Acad. Child Adolesc. Psychiatry.

[40] Milner A., Niedhammer I., Chastang J.-F., Spittal M., LaMontagne A. (2016). Job-exposure matrix for psychosocial job stressors: Results from the Household Income and Labour Dynamics in Australia Survey. PLoS ONE.

